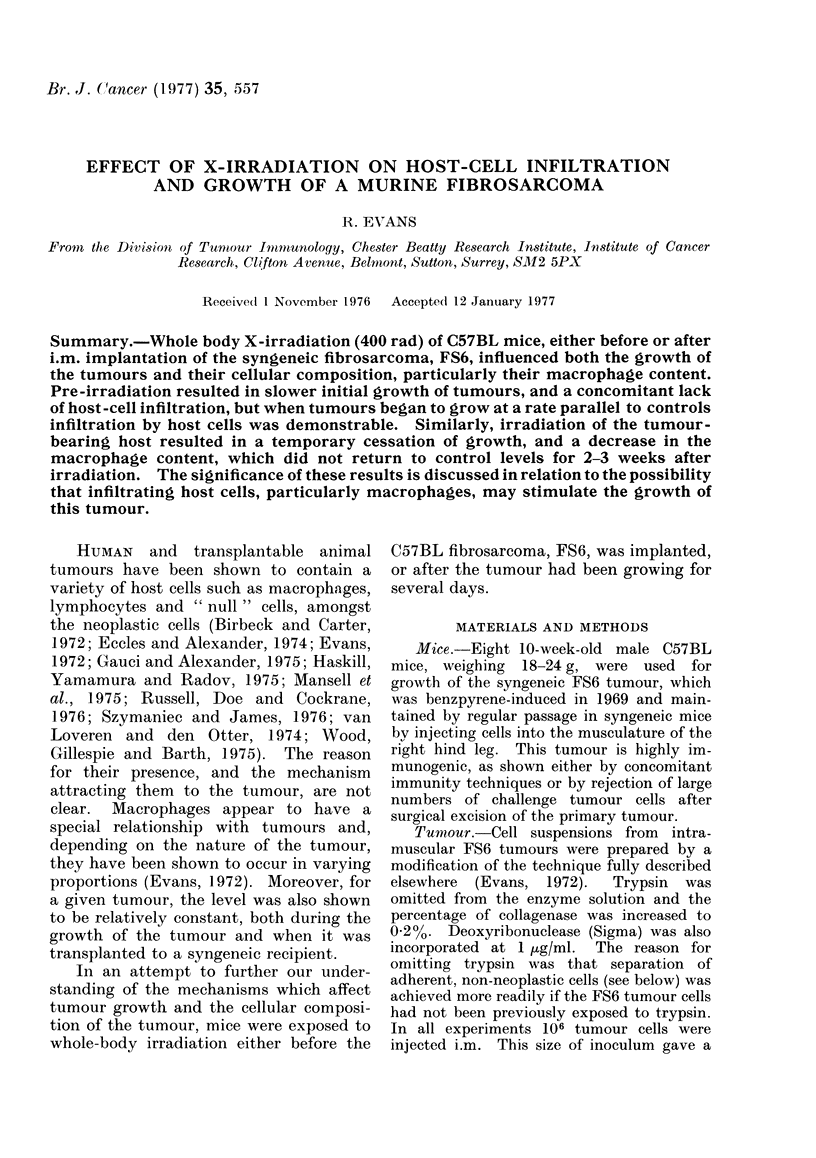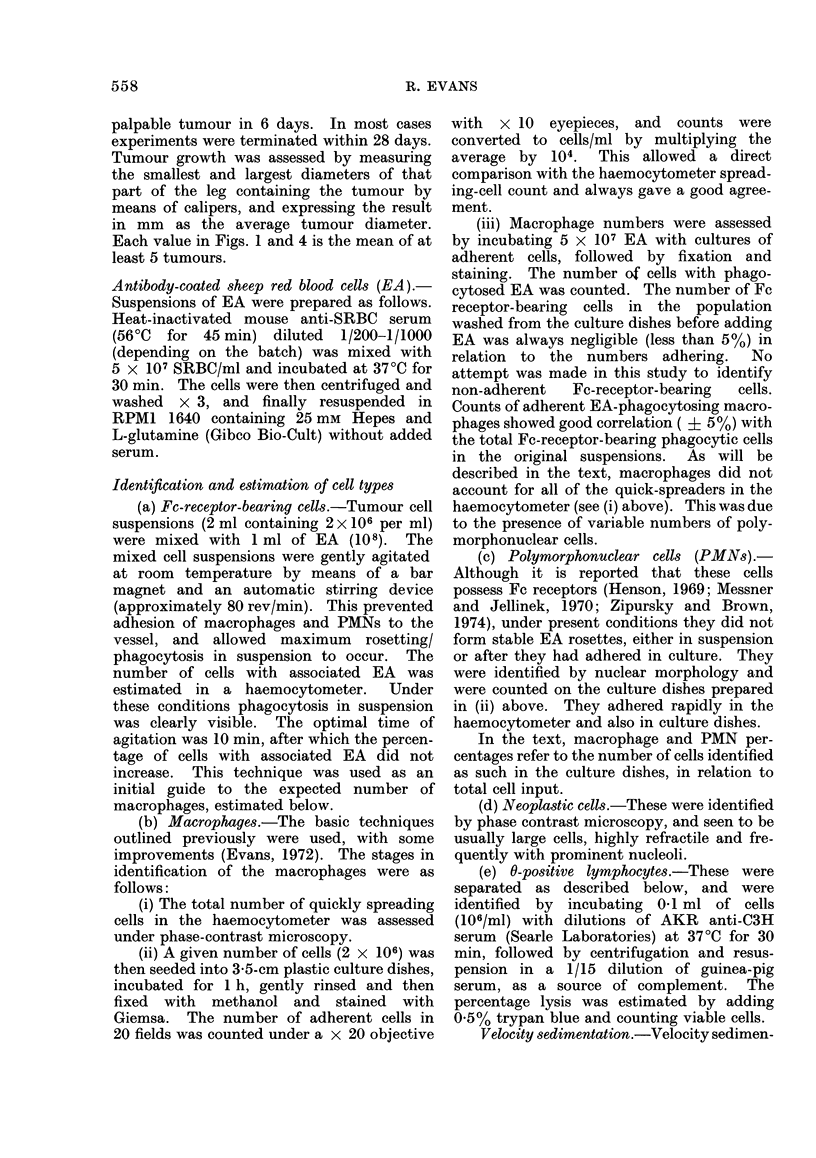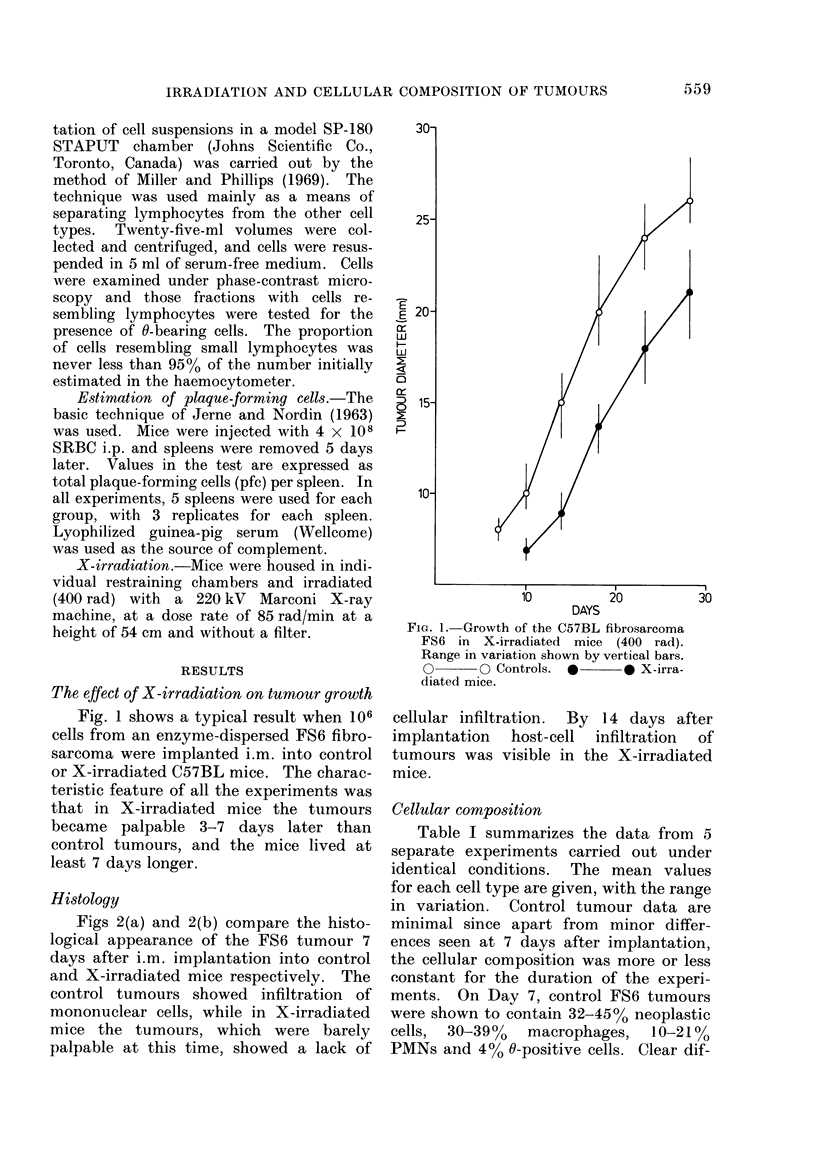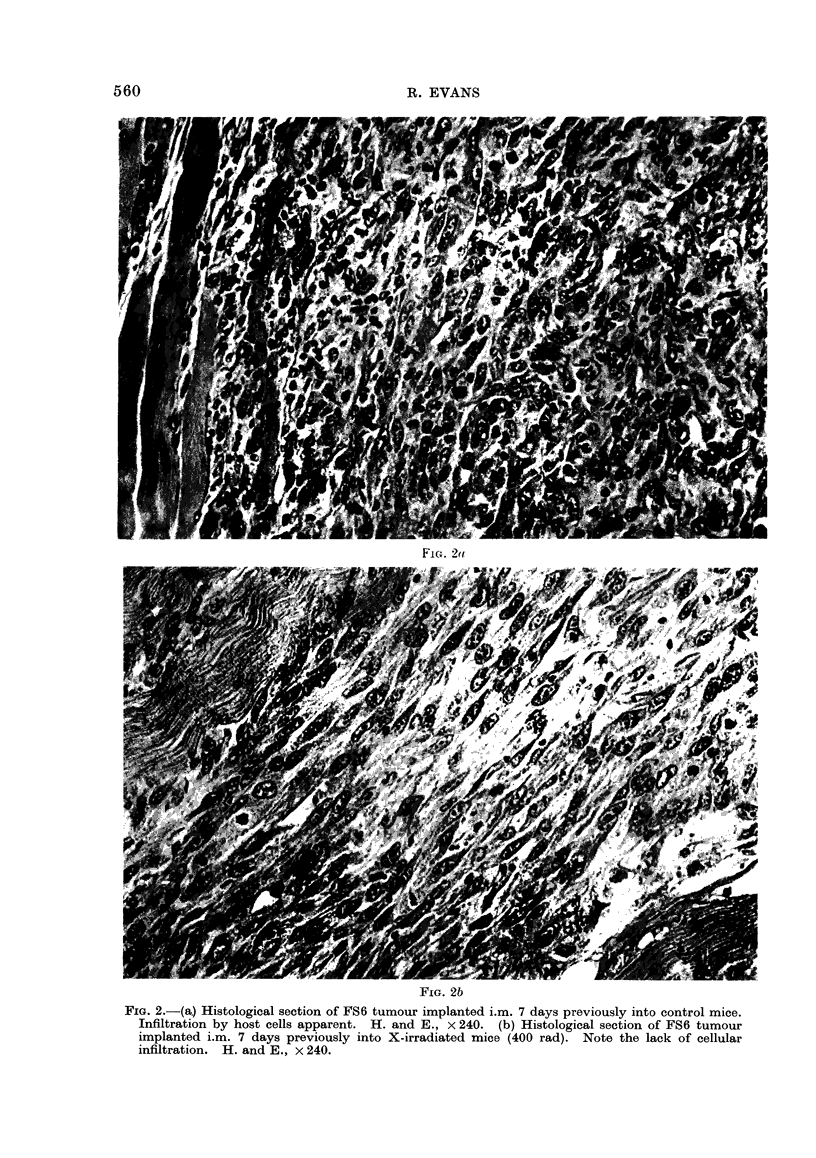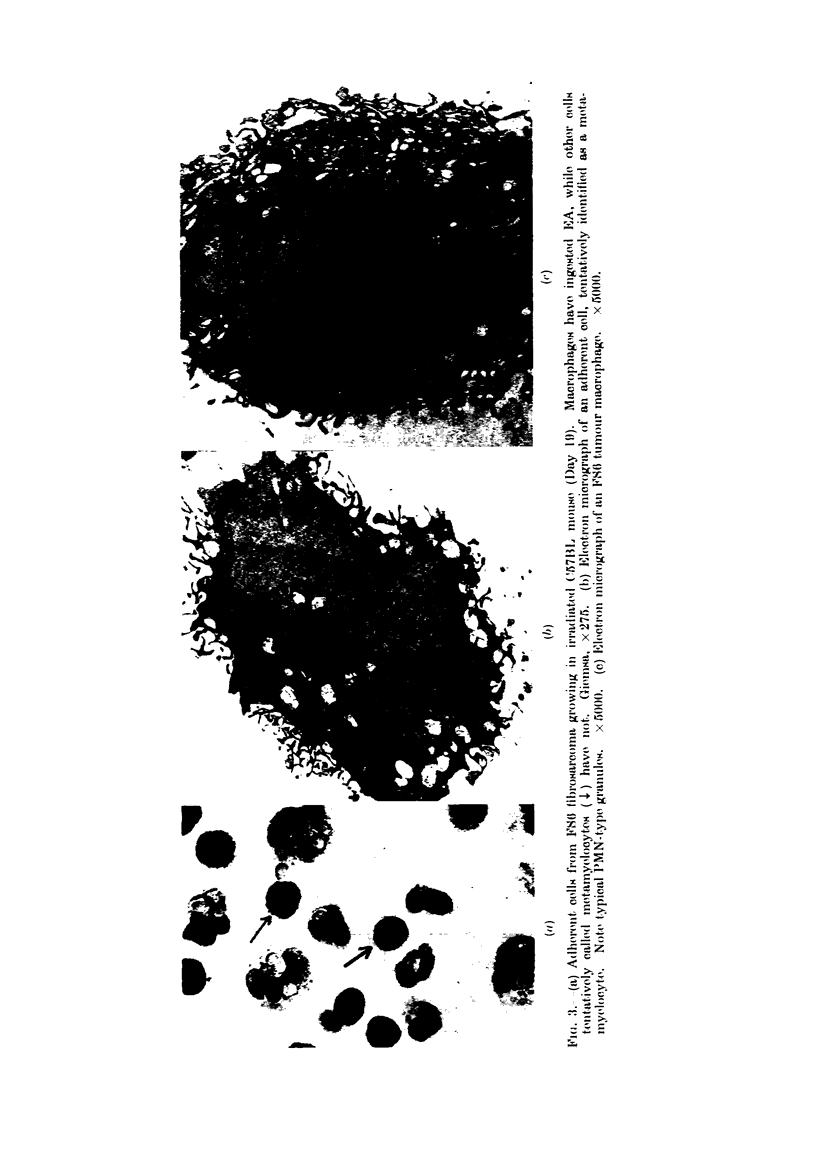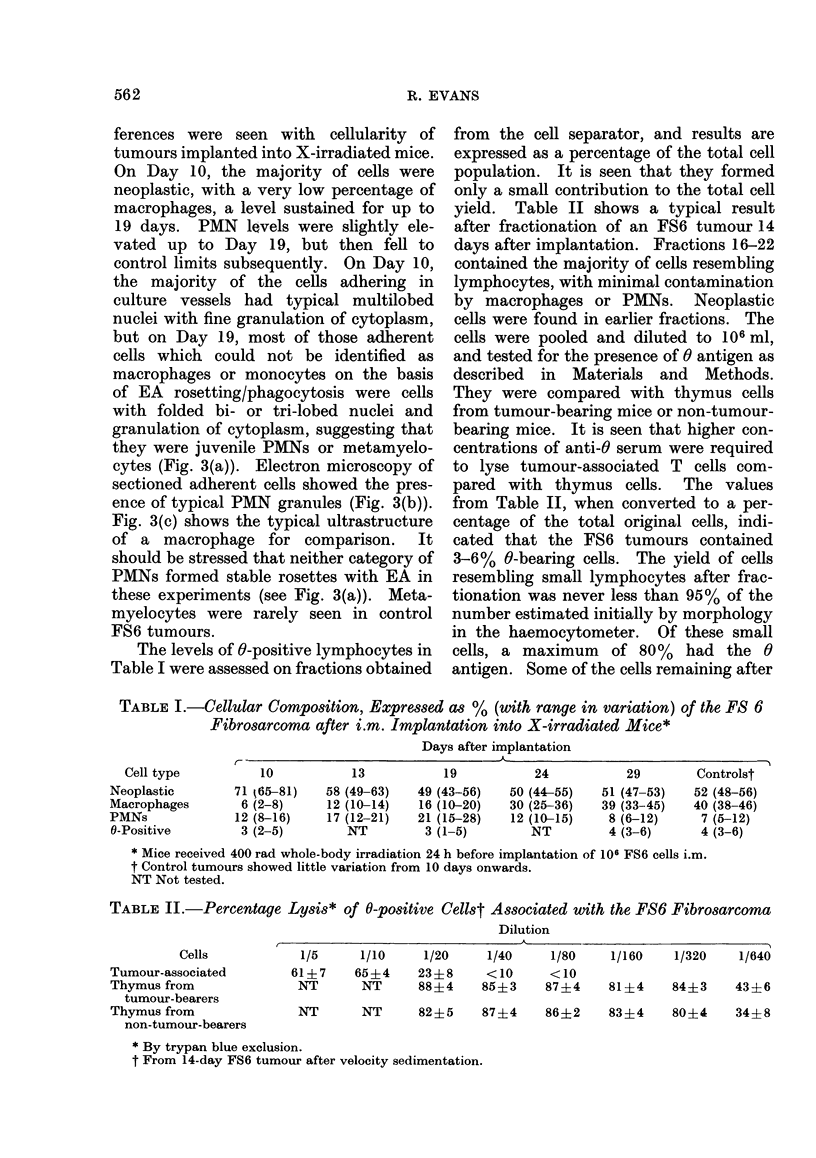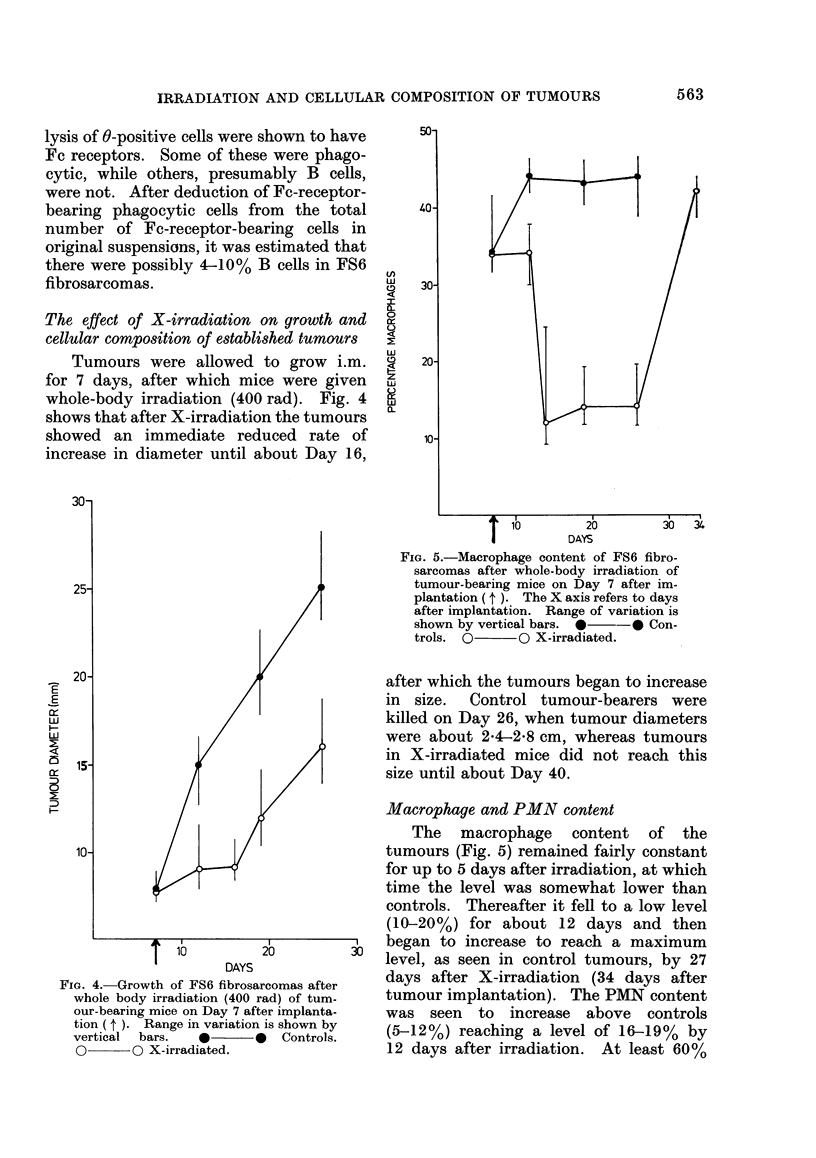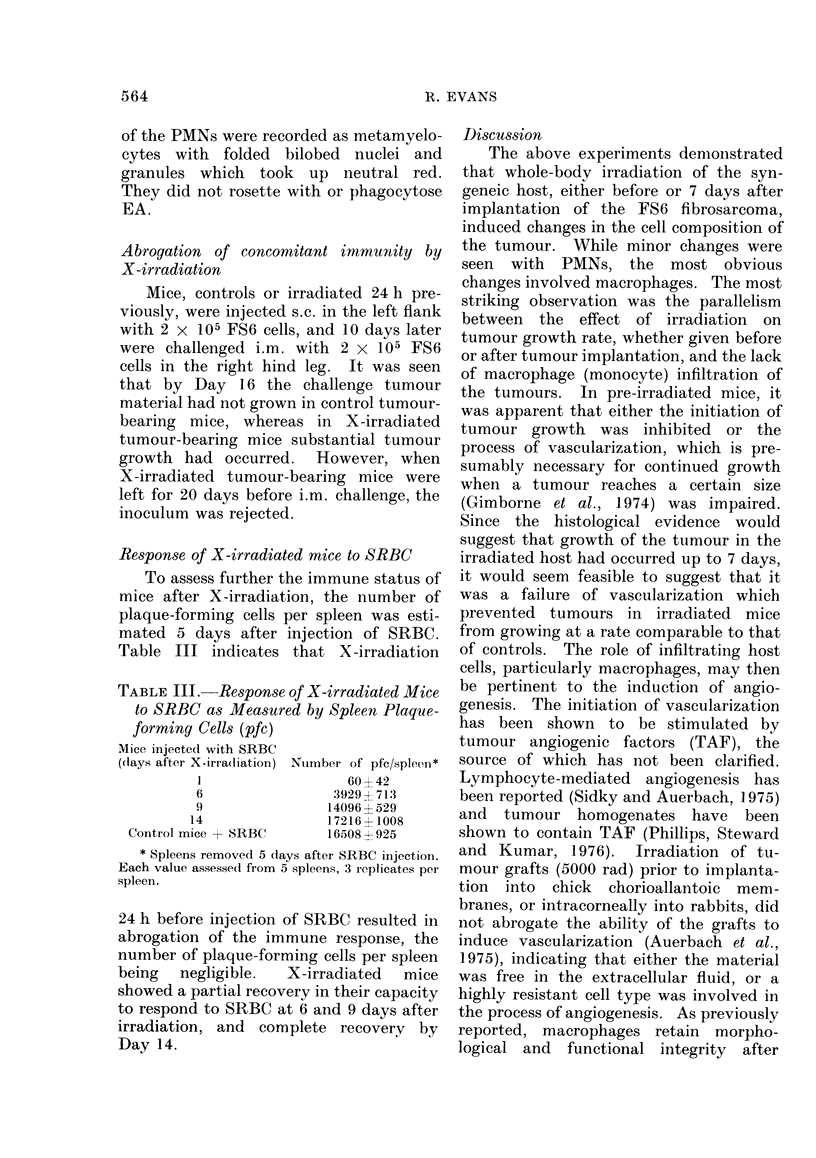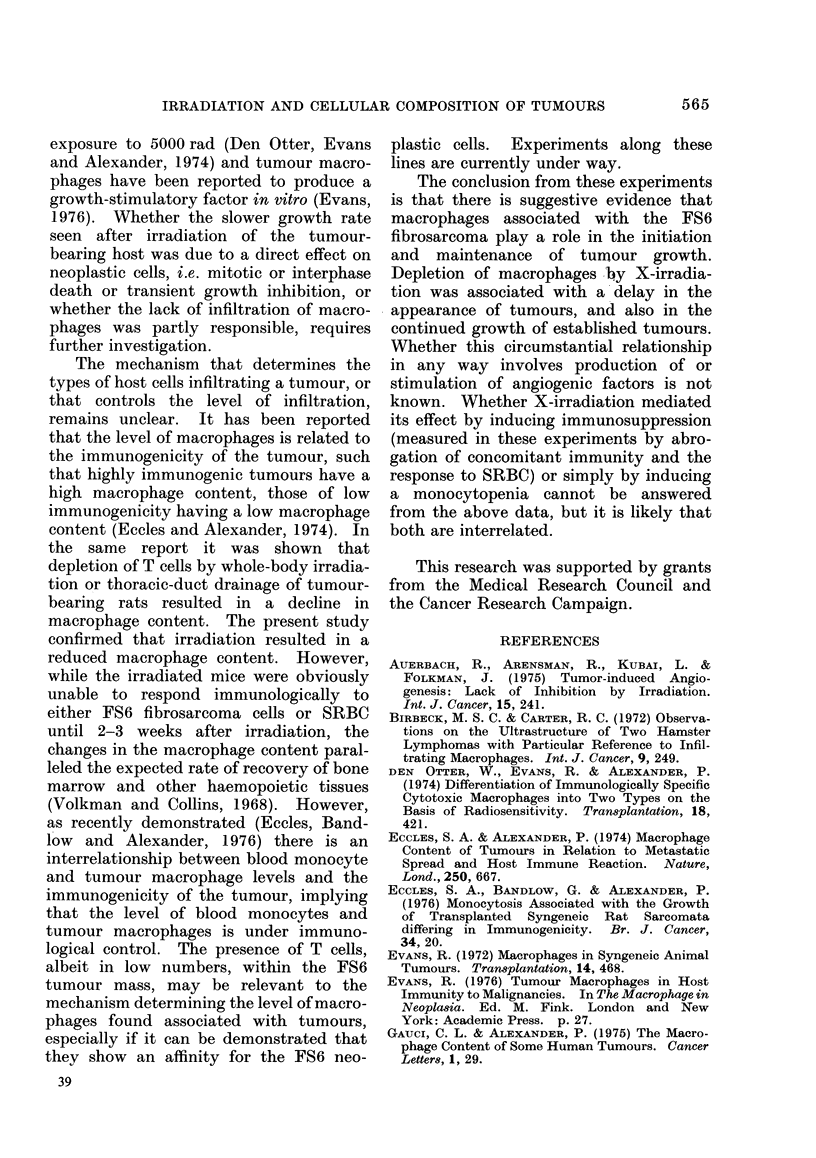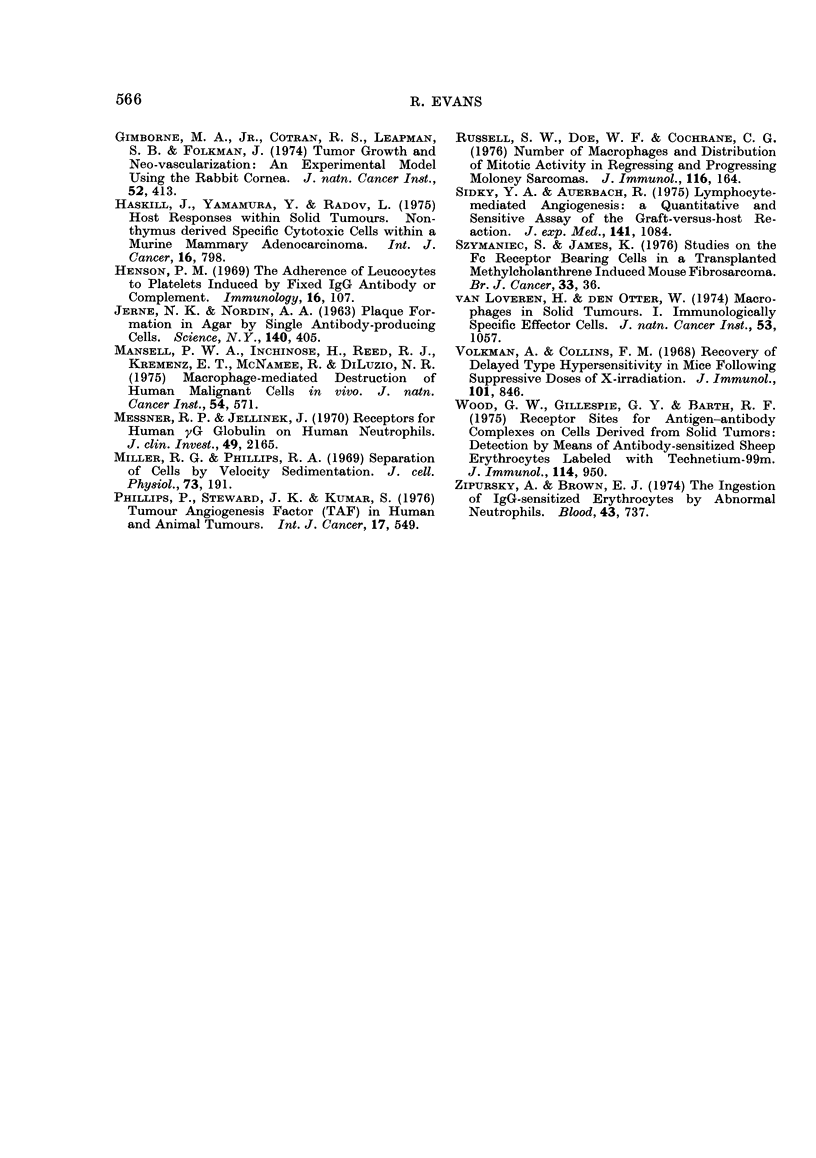# Effect of X-irradiation on host-cell infiltration and growth of a murine fibrosarcoma.

**DOI:** 10.1038/bjc.1977.89

**Published:** 1977-05

**Authors:** R. Evans

## Abstract

**Images:**


Br. J. Cancer (1977) 35, 557

### EFFECT OF X-IRRADIATION ON HOST-CELL INFILTRATION AND GROWTH OF A MURINE FIBROSARCOMA

R. EVANS

*From the Division of Tumour Immunology, Chester Beatty Research Institute, Institute of Cancer Research, Clifton Avenue, Belmont, Sutton, Surrey, SM2 5PX*

Received 1 November 1976 Accepted 12 January 1977

**Summary.—Whole body X-irradiation (400 rad) of C57BL mice, either before or after i.m. implantation of the syngeneic fibrosarcoma, FS6, influenced both the growth of the tumours and their cellular composition, particularly their macrophage content. Pre-irradiation resulted in slower initial growth of tumours, and a concomitant lack of host-cell infiltration, but when tumours began to grow at a rate parallel to controls infiltration by host cells was demonstrable. Similarly, irradiation of the tumour-bearing host resulted in a temporary cessation of growth, and a decrease in the macrophage content, which did not return to control levels for 2–3 weeks after irradiation. The significance of these results is discussed in relation to the possibility that infiltrating host cells, particularly macrophages, may stimulate the growth of this tumour.**

HUMAN and transplantable animal tumours have been shown to contain a variety of host cells such as macrophages, lymphocytes and " null " cells, amongst the neoplastic cells (Birbeck and Carter, 1972; Eccles and Alexander, 1974; Evans, 1972; Gauci and Alexander, 1975; Haskill, Yamamura and Radov, 1975; Mansell *et al.*, 1975; Russell, Doe and Cockrane, 1976; Szymaniec and James, 1976; van Loveren and den Otter, 1974; Wood, Gillespie and Barth, 1975). The reason for their presence, and the mechanism attracting them to the tumour, are not clear. Macrophages appear to have a special relationship with tumours and, depending on the nature of the tumour, they have been shown to occur in varying proportions (Evans, 1972). Moreover, for a given tumour, the level was also shown to be relatively constant, both during the growth of the tumour and when it was transplanted to a syngeneic recipient.

In an attempt to further our understanding of the mechanisms which affect tumour growth and the cellular composition of the tumour, mice were exposed to whole-body irradiation either before the C57BL fibrosarcoma, FS6, was implanted, or after the tumour had been growing for several days.

### MATERIALS AND METHODS

*Mice.*—Eight 10-week-old male C57BL mice, weighing 18–24 g, were used for growth of the syngeneic FS6 tumour, which was benzpyrene-induced in 1969 and maintained by regular passage in syngeneic mice by injecting cells into the musculature of the right hind leg. This tumour is highly immunogenic, as shown either by concomitant immunity techniques or by rejection of large numbers of challenge tumour cells after surgical excision of the primary tumour.

*Tumour.*—Cell suspensions from intramuscular FS6 tumours were prepared by a modification of the technique fully described elsewhere (Evans, 1972). Trypsin was omitted from the enzyme solution and the percentage of collagenase was increased to 0·2%. Deoxyribonuclease (Sigma) was also incorporated at 1 μg/ml. The reason for omitting trypsin was that separation of adherent, non-neoplastic cells (see below) was achieved more readily if the FS6 tumour cells had not been previously exposed to trypsin. In all experiments $10^6$ tumour cells were injected i.m. This size of inoculum gave a palpable tumour in 6 days. In most cases experiments were terminated within 28 days. Tumour growth was assessed by measuring the smallest and largest diameters of that part of the leg containing the tumour by means of calipers, and expressing the result in mm as the average tumour diameter. Each value in Figs. 1 and 4 is the mean of at least 5 tumours.

*Antibody-coated sheep red blood cells (EA)*.—Suspensions of EA were prepared as follows. Heat-inactivated mouse anti-SRBC serum (56°C for 45 min) diluted 1/200–1/1000 (depending on the batch) was mixed with $5 \times 10^7$ SRBC/ml and incubated at 37°C for 30 min. The cells were then centrifuged and washed $\times$ 3, and finally resuspended in RPM1 1640 containing 25 mM Hepes and L-glutamine (Gibco Bio-Cult) without added serum.

*Identification and estimation of cell types*

(a) *Fc-receptor-bearing cells*.—Tumour cell suspensions (2 ml containing $2 \times 10^6$ per ml) were mixed with 1 ml of EA ($10^8$). The mixed cell suspensions were gently agitated at room temperature by means of a bar magnet and an automatic stirring device (approximately 80 rev/min). This prevented adhesion of macrophages and PMNs to the vessel, and allowed maximum rosetting/phagocytosis in suspension to occur. The number of cells with associated EA was estimated in a haemocytometer. Under these conditions phagocytosis in suspension was clearly visible. The optimal time of agitation was 10 min, after which the percentage of cells with associated EA did not increase. This technique was used as an initial guide to the expected number of macrophages, estimated below.

(b) *Macrophages*.—The basic techniques outlined previously were used, with some improvements (Evans, 1972). The stages in identification of the macrophages were as follows:

(i) The total number of quickly spreading cells in the haemocytometer was assessed under phase-contrast microscopy.

(ii) A given number of cells ($2 \times 10^6$) was then seeded into 3·5-cm plastic culture dishes, incubated for 1 h, gently rinsed and then fixed with methanol and stained with Giemsa. The number of adherent cells in 20 fields was counted under a $\times$ 20 objective with $\times$ 10 eyepieces, and counts were converted to cells/ml by multiplying the average by $10^4$. This allowed a direct comparison with the haemocytometer spreading-cell count and always gave a good agreement.

(iii) Macrophage numbers were assessed by incubating $5 \times 10^7$ EA with cultures of adherent cells, followed by fixation and staining. The number of cells with phagocytosed EA was counted. The number of Fc receptor-bearing cells in the population washed from the culture dishes before adding EA was always negligible (less than 5%) in relation to the numbers adhering. No attempt was made in this study to identify non-adherent Fc-receptor-bearing cells. Counts of adherent EA-phagocytosing macrophages showed good correlation ( $\pm$ 5%) with the total Fc-receptor-bearing phagocytic cells in the original suspensions. As will be described in the text, macrophages did not account for all of the quick-spreaders in the haemocytometer (see (i) above). This was due to the presence of variable numbers of polymorphonuclear cells.

(c) *Polymorphonuclear cells (PMNs)*.—Although it is reported that these cells possess Fc receptors (Henson, 1969; Messner and Jellinek, 1970; Zipursky and Brown, 1974), under present conditions they did not form stable EA rosettes, either in suspension or after they had adhered in culture. They were identified by nuclear morphology and were counted on the culture dishes prepared in (ii) above. They adhered rapidly in the haemocytometer and also in culture dishes.

In the text, macrophage and PMN percentages refer to the number of cells identified as such in the culture dishes, in relation to total cell input.

(d) *Neoplastic cells*.—These were identified by phase contrast microscopy, and seen to be usually large cells, highly refractile and frequently with prominent nucleoli.

(e) *θ-positive lymphocytes*.—These were separated as described below, and were identified by incubating 0·1 ml of cells ($10^6$/ml) with dilutions of AKR anti-C3H serum (Searle Laboratories) at 37°C for 30 min, followed by centrifugation and resuspension in a 1/15 dilution of guinea-pig serum, as a source of complement. The percentage lysis was estimated by adding 0·5% trypan blue and counting viable cells.

*Velocity sedimentation*.—Velocity sedimentation of cell suspensions in a model SP-180 STAPUT chamber (Johns Scientific Co., Toronto, Canada) was carried out by the method of Miller and Phillips (1969). The technique was used mainly as a means of separating lymphocytes from the other cell types. Twenty-five-ml volumes were collected and centrifuged, and cells were resuspended in 5 ml of serum-free medium. Cells were examined under phase-contrast microscopy and those fractions with cells resembling lymphocytes were tested for the presence of $\theta$-bearing cells. The proportion of cells resembling small lymphocytes was never less than 95% of the number initially estimated in the haemocytometer.

*Estimation of plaque-forming cells.*—The basic technique of Jerne and Nordin (1963) was used. Mice were injected with $4 \times 10^8$ SRBC i.p. and spleens were removed 5 days later. Values in the test are expressed as total plaque-forming cells (pfc) per spleen. In all experiments, 5 spleens were used for each group, with 3 replicates for each spleen. Lyophilized guinea-pig serum (Wellcome) was used as the source of complement.

*X-irradiation.*—Mice were housed in individual restraining chambers and irradiated (400 rad) with a 220 kV Marconi X-ray machine, at a dose rate of 85 rad/min at a height of 54 cm and without a filter.

FIG. 1.—Growth of the C57BL fibrosarcoma FS6 in X-irradiated mice (400 rad). Range in variation shown by vertical bars. ○———○ Controls. ●———● X-irradiated mice.

### RESULTS

### *The effect of X-irradiation on tumour growth*

Fig. 1 shows a typical result when $10^6$ cells from an enzyme-dispersed FS6 fibrosarcoma were implanted i.m. into control or X-irradiated C57BL mice. The characteristic feature of all the experiments was that in X-irradiated mice the tumours became palpable 3–7 days later than control tumours, and the mice lived at least 7 days longer.

### *Histology*

Figs 2(a) and 2(b) compare the histological appearance of the FS6 tumour 7 days after i.m. implantation into control and X-irradiated mice respectively. The control tumours showed infiltration of mononuclear cells, while in X-irradiated mice the tumours, which were barely palpable at this time, showed a lack of cellular infiltration. By 14 days after implantation host-cell infiltration of tumours was visible in the X-irradiated mice.

### *Cellular composition*

Table I summarizes the data from 5 separate experiments carried out under identical conditions. The mean values for each cell type are given, with the range in variation. Control tumour data are minimal since apart from minor differences seen at 7 days after implantation, the cellular composition was more or less constant for the duration of the experiments. On Day 7, control FS6 tumours were shown to contain 32–45% neoplastic cells, 30–39% macrophages, 10–21% PMNs and 4% $\theta$-positive cells. Clear dif-

FIG. 2a

FIG. 2b

FIG. 2.—(a) Histological section of FS6 tumour implanted i.m. 7 days previously into control mice. Infiltration by host cells apparent. H. and E., ×240. (b) Histological section of FS6 tumour implanted i.m. 7 days previously into X-irradiated mice (400 rad). Note the lack of cellular infiltration. H. and E., ×240.

(a) (b) (c)

FIG. 3.—(a) Adherent cells from FS6 fibrosarcoma growing in irradiated C57BL mouse (Day 19). Macrophages have ingested EA, while other cells tentatively called metamyelocytes (↓) have not. Giemsa, ×275. (b) Electron micrograph of an adherent cell, tentatively identified as a metamyelocyte. Note typical PMN-type granules. ×5000. (c) Electron micrograph of an FS6 tumour macrophage. ×5000.

ferences were seen with cellularity of tumours implanted into X-irradiated mice. On Day 10, the majority of cells were neoplastic, with a very low percentage of macrophages, a level sustained for up to 19 days. PMN levels were slightly elevated up to Day 19, but then fell to control limits subsequently. On Day 10, the majority of the cells adhering in culture vessels had typical multilobed nuclei with fine granulation of cytoplasm, but on Day 19, most of those adherent cells which could not be identified as macrophages or monocytes on the basis of EA rosetting/phagocytosis were cells with folded bi- or tri-lobed nuclei and granulation of cytoplasm, suggesting that they were juvenile PMNs or metamyelocytes (Fig. 3(a)). Electron microscopy of sectioned adherent cells showed the presence of typical PMN granules (Fig. 3(b)). Fig. 3(c) shows the typical ultrastructure of a macrophage for comparison. It should be stressed that neither category of PMNs formed stable rosettes with EA in these experiments (see Fig. 3(a)). Metamyelocytes were rarely seen in control FS6 tumours.

The levels of $\theta$-positive lymphocytes in Table I were assessed on fractions obtained from the cell separator, and results are expressed as a percentage of the total cell population. It is seen that they formed only a small contribution to the total cell yield. Table II shows a typical result after fractionation of an FS6 tumour 14 days after implantation. Fractions 16–22 contained the majority of cells resembling lymphocytes, with minimal contamination by macrophages or PMNs. Neoplastic cells were found in earlier fractions. The cells were pooled and diluted to $10^6$ ml, and tested for the presence of $\theta$ antigen as described in Materials and Methods. They were compared with thymus cells from tumour-bearing mice or non-tumour-bearing mice. It is seen that higher concentrations of anti-$\theta$ serum were required to lyse tumour-associated T cells compared with thymus cells. The values from Table II, when converted to a percentage of the total original cells, indicated that the FS6 tumours contained 3–6% $\theta$-bearing cells. The yield of cells resembling small lymphocytes after fractionation was never less than 95% of the number estimated initially by morphology in the haemocytometer. Of these small cells, a maximum of 80% had the $\theta$ antigen. Some of the cells remaining after

TABLE I.—*Cellular Composition, Expressed as % (with range in variation) of the FS 6 Fibrosarcoma after i.m. Implantation into X-irradiated Mice**

| | Days after implantation | | | | | |
|---|---|---|---|---|---|---|
| Cell type | 10 | 13 | 19 | 24 | 29 | Controls† |
| Neoplastic | 71 (65–81) | 58 (49–63) | 49 (43–56) | 50 (44–55) | 51 (47–53) | 52 (48–56) |
| Macrophages | 6 (2–8) | 12 (10–14) | 16 (10–20) | 30 (25–36) | 39 (33–45) | 40 (38–46) |
| PMNs | 12 (8–16) | 17 (12–21) | 21 (15–28) | 12 (10–15) | 8 (6–12) | 7 (5–12) |
| $\theta$-Positive | 3 (2–5) | NT | 3 (1–5) | NT | 4 (3–6) | 4 (3–6) |

* Mice received 400 rad whole-body irradiation 24 h before implantation of $10^6$ FS6 cells i.m.
† Control tumours showed little variation from 10 days onwards.
NT Not tested.

TABLE II.—*Percentage Lysis* of $\theta$-positive Cells† Associated with the FS6 Fibrosarcoma*

| | Dilution | | | | | | | |
|---|---|---|---|---|---|---|---|---|
| Cells | 1/5 | 1/10 | 1/20 | 1/40 | 1/80 | 1/160 | 1/320 | 1/640 |
| Tumour-associated | 61±7 | 65±4 | 23±8 | <10 | <10 | | | |
| Thymus from tumour-bearers | NT | NT | 88±4 | 85±3 | 87±4 | 81±4 | 84±3 | 43±6 |
| Thymus from non-tumour-bearers | NT | NT | 82±5 | 87±4 | 86±2 | 83±4 | 80±4 | 34±8 |

* By trypan blue exclusion.
† From 14-day FS6 tumour after velocity sedimentation.

lysis of θ-positive cells were shown to have Fc receptors. Some of these were phagocytic, while others, presumably B cells, were not. After deduction of Fc-receptor-bearing phagocytic cells from the total number of Fc-receptor-bearing cells in original suspensions, it was estimated that there were possibly 4–10% B cells in FS6 fibrosarcomas.

*The effect of X-irradiation on growth and cellular composition of established tumours*

Tumours were allowed to grow i.m. for 7 days, after which mice were given whole-body irradiation (400 rad). Fig. 4 shows that after X-irradiation the tumours showed an immediate reduced rate of increase in diameter until about Day 16,

FIG. 4.—Growth of FS6 fibrosarcomas after whole body irradiation (400 rad) of tumour-bearing mice on Day 7 after implantation (↑). Range in variation is shown by vertical bars. ●——● Controls. ○——○ X-irradiated.

FIG. 5.—Macrophage content of FS6 fibrosarcomas after whole-body irradiation of tumour-bearing mice on Day 7 after implantation (↑). The X axis refers to days after implantation. Range of variation is shown by vertical bars. ●——● Controls. ○——○ X-irradiated.

after which the tumours began to increase in size. Control tumour-bearers were killed on Day 26, when tumour diameters were about 2·4–2·8 cm, whereas tumours in X-irradiated mice did not reach this size until about Day 40.

*Macrophage and PMN content*

The macrophage content of the tumours (Fig. 5) remained fairly constant for up to 5 days after irradiation, at which time the level was somewhat lower than controls. Thereafter it fell to a low level (10–20%) for about 12 days and then began to increase to reach a maximum level, as seen in control tumours, by 27 days after X-irradiation (34 days after tumour implantation). The PMN content was seen to increase above controls (5–12%) reaching a level of 16–19% by 12 days after irradiation. At least 60%

of the PMNs were recorded as metamyelocytes with folded bilobed nuclei and granules which took up neutral red. They did not rosette with or phagocytose EA.

*Abrogation of concomitant immunity by X-irradiation*

Mice, controls or irradiated 24 h previously, were injected s.c. in the left flank with $2 \times 10^5$ FS6 cells, and 10 days later were challenged i.m. with $2 \times 10^5$ FS6 cells in the right hind leg. It was seen that by Day 16 the challenge tumour material had not grown in control tumour-bearing mice, whereas in X-irradiated tumour-bearing mice substantial tumour growth had occurred. However, when X-irradiated tumour-bearing mice were left for 20 days before i.m. challenge, the inoculum was rejected.

*Response of X-irradiated mice to SRBC*

To assess further the immune status of mice after X-irradiation, the number of plaque-forming cells per spleen was estimated 5 days after injection of SRBC. Table III indicates that X-irradiation 24 h before injection of SRBC resulted in abrogation of the immune response, the number of plaque-forming cells per spleen being negligible. X-irradiated mice showed a partial recovery in their capacity to respond to SRBC at 6 and 9 days after irradiation, and complete recovery by Day 14.

TABLE III.—*Response of X-irradiated Mice to SRBC as Measured by Spleen Plaque-forming Cells (pfc)*

| Mice injected with SRBC (days after X-irradiation) | Number of pfc/spleen* |
|---|---|
| 1 | 60 ± 42 |
| 6 | 3929 ± 713 |
| 9 | 14096 ± 529 |
| 14 | 17216 ± 1008 |
| Control mice + SRBC | 16508 ± 925 |

* Spleens removed 5 days after SRBC injection. Each value assessed from 5 spleens, 3 replicates per spleen.

*Discussion*

The above experiments demonstrated that whole-body irradiation of the syngeneic host, either before or 7 days after implantation of the FS6 fibrosarcoma, induced changes in the cell composition of the tumour. While minor changes were seen with PMNs, the most obvious changes involved macrophages. The most striking observation was the parallelism between the effect of irradiation on tumour growth rate, whether given before or after tumour implantation, and the lack of macrophage (monocyte) infiltration of the tumours. In pre-irradiated mice, it was apparent that either the initiation of tumour growth was inhibited or the process of vascularization, which is presumably necessary for continued growth when a tumour reaches a certain size (Gimborne *et al.*, 1974) was impaired. Since the histological evidence would suggest that growth of the tumour in the irradiated host had occurred up to 7 days, it would seem feasible to suggest that it was a failure of vascularization which prevented tumours in irradiated mice from growing at a rate comparable to that of controls. The role of infiltrating host cells, particularly macrophages, may then be pertinent to the induction of angiogenesis. The initiation of vascularization has been shown to be stimulated by tumour angiogenic factors (TAF), the source of which has not been clarified. Lymphocyte-mediated angiogenesis has been reported (Sidky and Auerbach, 1975) and tumour homogenates have been shown to contain TAF (Phillips, Steward and Kumar, 1976). Irradiation of tumour grafts (5000 rad) prior to implantation into chick chorioallantoic membranes, or intracorneally into rabbits, did not abrogate the ability of the grafts to induce vascularization (Auerbach *et al.*, 1975), indicating that either the material was free in the extracellular fluid, or a highly resistant cell type was involved in the process of angiogenesis. As previously reported, macrophages retain morphological and functional integrity after exposure to 5000 rad (Den Otter, Evans and Alexander, 1974) and tumour macrophages have been reported to produce a growth-stimulatory factor *in vitro* (Evans, 1976). Whether the slower growth rate seen after irradiation of the tumour-bearing host was due to a direct effect on neoplastic cells, *i.e.* mitotic or interphase death or transient growth inhibition, or whether the lack of infiltration of macrophages was partly responsible, requires further investigation.

The mechanism that determines the types of host cells infiltrating a tumour, or that controls the level of infiltration, remains unclear. It has been reported that the level of macrophages is related to the immunogenicity of the tumour, such that highly immunogenic tumours have a high macrophage content, those of low immunogenicity having a low macrophage content (Eccles and Alexander, 1974). In the same report it was shown that depletion of T cells by whole-body irradiation or thoracic-duct drainage of tumour-bearing rats resulted in a decline in macrophage content. The present study confirmed that irradiation resulted in a reduced macrophage content. However, while the irradiated mice were obviously unable to respond immunologically to either FS6 fibrosarcoma cells or SRBC until 2–3 weeks after irradiation, the changes in the macrophage content paralleled the expected rate of recovery of bone marrow and other haemopoietic tissues (Volkman and Collins, 1968). However, as recently demonstrated (Eccles, Bandlow and Alexander, 1976) there is an interrelationship between blood monocyte and tumour macrophage levels and the immunogenicity of the tumour, implying that the level of blood monocytes and tumour macrophages is under immunological control. The presence of T cells, albeit in low numbers, within the FS6 tumour mass, may be relevant to the mechanism determining the level of macrophages found associated with tumours, especially if it can be demonstrated that they show an affinity for the FS6 neoplastic cells. Experiments along these lines are currently under way.

The conclusion from these experiments is that there is suggestive evidence that macrophages associated with the FS6 fibrosarcoma play a role in the initiation and maintenance of tumour growth. Depletion of macrophages by X-irradiation was associated with a delay in the appearance of tumours, and also in the continued growth of established tumours. Whether this circumstantial relationship in any way involves production of or stimulation of angiogenic factors is not known. Whether X-irradiation mediated its effect by inducing immunosuppression (measured in these experiments by abrogation of concomitant immunity and the response to SRBC) or simply by inducing a monocytopenia cannot be answered from the above data, but it is likely that both are interrelated.

This research was supported by grants from the Medical Research Council and the Cancer Research Campaign.

### REFERENCES

AUERBACH, R., ARENSMAN, R., KUBAI, L. & FOLKMAN, J. (1975) Tumor-induced Angiogenesis: Lack of Inhibition by Irradiation. *Int. J. Cancer*, **15**, 241.

BIRBECK, M. S. C. & CARTER, R. C. (1972) Observations on the Ultrastructure of Two Hamster Lymphomas with Particular Reference to Infiltrating Macrophages. *Int. J. Cancer*, **9**, 249.

DEN OTTER, W., EVANS, R. & ALEXANDER, P. (1974) Differentiation of Immunologically Specific Cytotoxic Macrophages into Two Types on the Basis of Radiosensitivity. *Transplantation*, **18**, 421.

ECCLES, S. A. & ALEXANDER, P. (1974) Macrophage Content of Tumours in Relation to Metastatic Spread and Host Immune Reaction. *Nature, Lond.*, **250**, 667.

ECCLES, S. A., BANDLOW, G. & ALEXANDER, P. (1976) Monocytosis Associated with the Growth of Transplanted Syngeneic Rat Sarcomata differing in Immunogenicity. *Br. J. Cancer*, **34**, 20.

EVANS, R. (1972) Macrophages in Syngeneic Animal Tumours. *Transplantation*, **14**, 468.

EVANS, R. (1976) Tumour Macrophages in Host Immunity to Malignancies. In *The Macrophage in Neoplasia*. Ed. M. Fink. London and New York: Academic Press. p. 27.

GAUCI, C. L. & ALEXANDER, P. (1975) The Macrophage Content of Some Human Tumours. *Cancer Letters*, **1**, 29.

GIMBORNE, M. A., JR., COTRAN, R. S., LEAPMAN, S. B. & FOLKMAN, J. (1974) Tumor Growth and Neo-vascularization: An Experimental Model Using the Rabbit Cornea. *J. natn. Cancer Inst.*, **52**, 413.

HASKILL, J., YAMAMURA, Y. & RADOV, L. (1975) Host Responses within Solid Tumours. Non-thymus derived Specific Cytotoxic Cells within a Murine Mammary Adenocarcinoma. *Int. J. Cancer*, **16**, 798.

HENSON, P. M. (1969) The Adherence of Leucocytes to Platelets Induced by Fixed IgG Antibody or Complement. *Immunology*, **16**, 107.

JERNE, N. K. & NORDIN, A. A. (1963) Plaque Formation in Agar by Single Antibody-producing Cells. *Science, N.Y.*, **140**, 405.

MANSELL, P. W. A., INCHINOSE, H., REED, R. J., KREMENZ, E. T., MCNAMEE, R. & DILUZIO, N. R. (1975) Macrophage-mediated Destruction of Human Malignant Cells *in vivo*. *J. natn. Cancer Inst.*, **54**, 571.

MESSNER, R. P. & JELLINEK, J. (1970) Receptors for Human $\gamma$G Globulin on Human Neutrophils. *J. clin. Invest.*, **49**, 2165.

MILLER, R. G. & PHILLIPS, R. A. (1969) Separation of Cells by Velocity Sedimentation. *J. cell. Physiol.*, **73**, 191.

PHILLIPS, P., STEWARD, J. K. & KUMAR, S. (1976) Tumour Angiogenesis Factor (TAF) in Human and Animal Tumours. *Int. J. Cancer*, **17**, 549.

RUSSELL, S. W., DOE, W. F. & COCHRANE, C. G. (1976) Number of Macrophages and Distribution of Mitotic Activity in Regressing and Progressing Moloney Sarcomas. *J. Immunol.*, **116**, 164.

SIDKY, Y. A. & AUERBACH, R. (1975) Lymphocyte-mediated Angiogenesis: a Quantitative and Sensitive Assay of the Graft-versus-host Reaction. *J. exp. Med.*, **141**, 1084.

SZYMANIEC, S. & JAMES, K. (1976) Studies on the Fc Receptor Bearing Cells in a Transplanted Methylcholanthrene Induced Mouse Fibrosarcoma. *Br. J. Cancer*, **33**, 36.

VAN LOVEREN, H. & DEN OTTER, W. (1974) Macrophages in Solid Tumours. I. Immunologically Specific Effector Cells. *J. natn. Cancer Inst.*, **53**, 1057.

VOLKMAN, A. & COLLINS, F. M. (1968) Recovery of Delayed Type Hypersensitivity in Mice Following Suppressive Doses of X-irradiation. *J. Immunol.*, **101**, 846.

WOOD, G. W., GILLESPIE, G. Y. & BARTH, R. F. (1975) Receptor Sites for Antigen–antibody Complexes on Cells Derived from Solid Tumors: Detection by Means of Antibody-sensitized Sheep Erythrocytes Labeled with Technetium-99m. *J. Immunol.*, **114**, 950.

ZIPURSKY, A. & BROWN, E. J. (1974) The Ingestion of IgG-sensitized Erythrocytes by Abnormal Neutrophils. *Blood*, **43**, 737.